# Efficacy of acupuncture in animal models of various ovarian dysfunctions: a systematic review and meta-analysis

**DOI:** 10.3389/fmed.2024.1348884

**Published:** 2024-06-20

**Authors:** Yuemeng Zhao, Ying Lan, Liying Liu, Jianheng Hao, Haijun Wang, Laixi Ji

**Affiliations:** ^1^Acupuncture and Tuina School, Chengdu University of Traditional Chinese Medicine, Chengdu, Sichuan Province, China; ^2^Prevention and Treatment Center Department, Chengdu University of Traditional Chinese Medicine Affiliated Hospital, Chengdu, Sichuan Province, China; ^3^Acupuncture and Tuina School, Shanxi University of Traditional Chinese Medicine, Taiyuan, Shanxi Province, China

**Keywords:** polycystic ovary syndrome, premature ovarian failure, perimenopausal syndrome, premature ovarian insufficiency, acupuncture, electroacupuncture, ovarian function, animal experiments

## Abstract

**Objective:**

This study aims to assess the comprehensive and integrated modulatory effects of acupuncture and electroacupuncture on various ovarian dysfunctions.

**Methods:**

We systematically searched for articles on animal experiments related to polycystic ovary syndrome (PCOS), premature ovarian failure (POF), premature ovarian insufficiency (POI), and perimenopausal syndrome (PMS) across multiple databases, including PubMed, Web of Science, Cochrane Library, Embase, and four Chinese language databases. The search covered the period from inception to November 2023. We conducted a comparative analysis between the acupuncture group and the model group (untreated) based on eligible literature. Our primary outcomes encompassed serum sex hormones (Luteinizing hormone, Follicle-stimulating hormone, Testosterone, Estradiol, Progesterone, and Anti-Müllerian hormone) and ovarian weight. Dichotomous data were synthesized to establish the relative risk (RR) of notable post-treatment improvement, while continuous data were pooled to determine the standardized mean difference (SMD) in post-treatment scores between the groups. Statistical analyses, including sensitivity analysis, Egger's test, and the trim-and-fill method, were executed using Stata 15.0 software.

**Results:**

The meta-analysis encompassed 29 articles involving a total of 623 rats. In comparison to rat models of PCOS, the experimental group exhibited a reduction in serum levels of LH, T and LH/FSH ratio. However, no statistically significant differences were observed in AMH, FSH, E_2_ levels, and ovarian weight between the two groups. In the ovarian hypoplasia model rats, both acupuncture and electroacupuncture interventions were associated with an increase in E_2_ levels. However, the levels of LH and FSH did not exhibit a significant difference between the two groups.

**Conclusions:**

Acupuncture or electroacupuncture facilitates the restoration of ovarian function primarily through the modulation of serum sex hormones, exerting regulatory effects across various types of ovarian dysfunction disorders.

**Systematic review registration:**

https://www.crd.york.ac.uk/prospero/display_record.php?ID=CRD42022316279

## 1 Introduction

Ovarian dysfunction, primarily attributed to endocrine disorders, manifests as menstrual irregularities and, in severe cases, infertility (1–5). In the last few decades, hormone replacement therapy (HRT) has been employed as an intervention for individuals grappling with ovarian dysfunction. Nevertheless, prolonged usage of HRT is associated with heightened risks of breast cancer, coronary heart disease, and venous thromboembolism. Furthermore, its efficacy in enhancing clinical pregnancy rates remains insubstantial (6, 7). Women of reproductive age frequently encounter the development of polycystic ovaries attributed to hormonal imbalances, resulting in delayed follicular development (8, 9). Additionally, stress and various factors affecting ovarian function can contribute to conditions such as diminished ovarian reserve (DOR), premature ovarian insufficiency (POI), and even premature ovarian failure (POF) (10). As women age, ovarian function naturally diminishes, leading to the onset of perimenopausal syndrome (PMS) and significantly impacting their daily lives (11, 12). The array of ovarian dysfunctions mentioned earlier constitutes factors associated with endocrine disruptions in women, resulting in compromised oocyte quality, diminished fertility, and premature aging.

Presently, acupuncture stands as a widely utilized intervention for addressing abnormal ovarian function, attributed to its well-tolerated side effects (13, 14). Acupuncture has been found to improve ovarian function, promote ovulation, relieve pain, ease emotional anxiety, and even delay diminished ovarian function (15–20). Extensive research has indicated that acupuncture not only enhances ovarian function and fosters ovulation but also provides relief from pain, mitigates emotional anxiety, and has the potential to postpone diminished ovarian function (21). Animal experiments and the subsequent integrated data analysis derived from these experiments can, to a certain extent, yield valuable a priori information. This information proves instrumental in guiding experimental or trial design and, in some cases, even informs clinical decision-making. The meta-analysis of preclinical animal studies plays a pivotal role in translational medicine, contributing to the development of more precise medical treatments (22, 23). While animal experiments offer valuable insights, their limitations include a low repetition rate of results. In an effort to delve further into the efficacy of acupuncture and minimize unnecessary attrition of animals in experiments, this study strategically categorized the PCOS animal model as a high-response group for ovarian function, while DOR, POI, POF, and PMS were collectively considered a low-response group for ovarian function. The aim was to investigate the effectiveness of acupuncture as a standalone intervention across different abnormal ovarian functions, providing a precise theoretical foundation for the acupuncture treatment of ovarian dysfunction diseases. Notably, this study protocol has been prospectively registered with Prospero (CRD42022316279).

## 2 Materials and methods

### 2.1 Search strategy

The search spanned from the inception of databases until November 2023, covering the Chinese National Knowledge Infrastructure (CNKI), Wanfang Data (WFDB), VIP Information Database, China Biology Medicine (CBM Disc), as well as international databases including PubMed, Embase, Cochrane Library, and Web of Science. Employing a combination of subject terms and free words, the search aimed to comprehensively capture relevant literature. Additionally, a manual search for references in pertinent literature and reviews was conducted (refer to Appendix 1) to ensure a thorough examination and avoidance of any potential omissions.

### 2.2 Inclusion criteria

The research was considered eligible if it met the following criteria:

1. Female Sprague Dawley (SD) rats were utilized as animal models for PCOS, POF, POI, DOR, and PMS.
2. Implementation of either simple acupuncture or electroacupuncture as the intervention.
3. Formation of experimental groups comprising both the model and acupuncture cohorts, with the control group consisting solely of the model group. In instances where included articles featured more than two groups, only the acupuncture and model groups were retained.
4. Assessment encompassing at least one of the following outcome indicators: luteinizing hormone (LH), follicle-stimulating hormone (FSH), testosterone (T), estradiol (E_2_), anti-Müllerian hormone (AMH), LH/FSH ratio, ovarian weight (OW), ovarian volume (OV), and follicle number (FN). Notably, all hormone tests were conducted using serum samples.

### 2.3 Exclusion criteria

The research that satisfied any of the following conditions are excluded:

1. Disruption of ovarian tissue morphology during the modeling process.
2. Utilization of acupuncture or electroacupuncture as a pre-treatment intervention before or during the construction of animal models.
3. In cases where data were duplicated across multiple instances, only the complete study was considered.
4. Studies lacking sufficient data were also excluded.

### 2.4 Selection of studies

Two reviewers (W.H.J. and L.L.Y.) independently performed literature screening, data extraction, and quality assessment for the trials. In cases of disagreement, a third party (Z.Y.M.) was consulted, and a consensus was reached. Titles and abstracts were meticulously reviewed, with articles not meeting the eligibility criteria being excluded. In instances of missing data, attempts were made to acquire the necessary information by reaching out to the corresponding authors via email. For articles presenting data solely in image format, two independent authors (L.Y.) utilized the webplot digitizer (https://apps.automeris.io/wpd/) to extract mean difference (MD) and standard deviation (SD) values from the images. The extracted data were cross-checked, and any disagreements were resolved through consultation with a third party (J.L.X. and L.Y.).

The characteristics and general information were systematically extracted and organized in tabular form. The extracted data encompassed details such as authors, publication date, animal species, intervention methods, modeling techniques, and study outcomes.

### 2.5 Bias risk and quality evaluation

The chosen studies underwent evaluation based on the guidelines outlined in the Animal Research: Reporting of *In Vivo* Experiments (ARRIVE) protocol (24, 25). To gauge the quality of each study, a ratio of the obtained score to the maximum possible score was calculated. This process resulted in three distinct quality intervals: studies scoring between 0.8–1 were categorized as “excellent,” those with scores between 0.5–0.8 were deemed “average,” while scores below 0.5 were categorized as “poor” (21, 26, 27).

The risk of bias in the selected studies was assessed using the Systematic Review Center for Laboratory Animal Experimentation (SYRCLE) tool (28). Each item within the tool was categorized as “yes,” “no,” or “unclear” to assign scores to the respective articles. Studies with at least one item identified as having a high risk of bias were classified as having an overall high risk of bias. Those with unclear risk of bias for at least one item were considered to be at an unclear risk of bias, while studies demonstrating low risk of bias across all items were rated as having a low risk of bias.

Two authors (Z.Y.M. and H.J.H.) independently conducted the quality and risk of bias assessments. Any discrepancies were resolved through discussion or, when necessary, by seeking the judgment of a third expert (L.Y.) until a consensus was reached.

### 2.6 Statistical analysis

In this study, serum sex hormone levels, ovarian weight, ovarian volume, and the number of follicles in both the disease and intervention groups were treated as continuous data in comparison to the control group. Standardized mean difference (SMD) was employed to evaluate the outcome indicators. The meta-analysis for each serum sex hormone level and ovarian morphology was conducted using Stata 15.0, with a confidence interval (CI) set at 95%.

If the *P* > 0.10 and *I*^2^ < 50%, indicating minimal heterogeneity among studies, the fixed-effects model is selected. Conversely, if these conditions are not met, the random-effects model is employed. Due to insufficient data on the main factors, we refrained from conducting subgroup analyses to investigate heterogeneity at this stage (29). However, we intend to perform future subgroup analyses, exploring variables such as different acupuncture protocols, shorter (≤2 weeks) or longer (>2 weeks) durations, and variations in animal model species.

For outcome indices with 10 or more included studies, we will conduct sensitivity analyses to explore the causes of heterogeneity. Additionally, Egger's method will be employed to test for publication bias. If the *P* < 0.05, indicating the presence of publication bias, appropriate measures, such as trimming or adjustments, will be considered to address the identified bias.

## 3 Results

### 3.1 Results of the search

**Figure 1:**
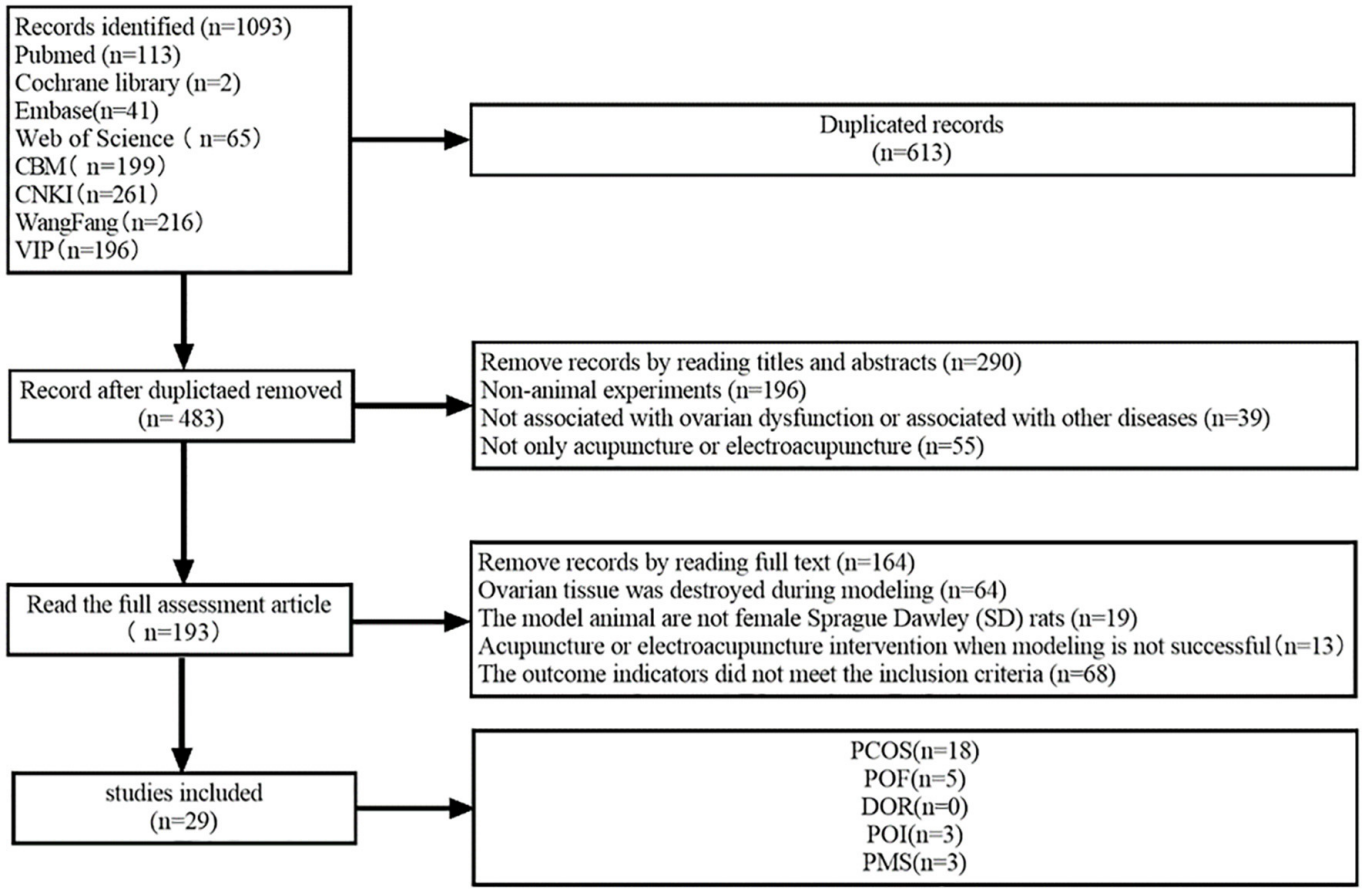
Flowchart of literature search.

A total of 1,093 publications were initially identified. Following rigorous selection criteria, 29 articles, encompassing a total of 623 rats, were ultimately included in the present meta-analysis. The distribution of these studies comprised 18 articles focused on PCOS, five on POF, three on PMS, zero on DOR and an additional three on PMS (Figure 1).

### 3.2 Characteristics of included studies

Twelve studies employed manual acupuncture, while 17 studies utilized electroacupuncture as the intervention method. Ovarian function was assessed through the collection of specimens, encompassing both serum samples and ovarian tissue. Detailed baseline characteristics of the included studies are provided in Table 1.

**Table 1:** General characteristics of the included studies.

**Study**	**Animal species**	**Disease model**	**Modeling drug**	**Intervention**	**Number of interventions**	**Sampling time**	**Measurement index**	**Acupoint**	**References**
Xu 2019	SD	PCOS	CAS	EA	28	Interestrus	LH, FSH, AMH, NF, LH/FSH	ST36, SP6, CV4	(30)
Xu 2018	SD	PCOS	CAS	EA	14	Interestrus	E_2_, T, OW	ST36, SP6, CV4	(31)
Sun 2013	SD	PCOS	Letrozole	EA	14	Unclear	LH, FSH, E_2_, T, OW	CV4, CV3	(32)
Shen 2021	SD	PCOS	Letrozole	EA	15	Unclear	LH, FSH, E_2_, T, NF	GB26	(33)
Li 2012	SD	PCOS	DHEA	EA	30	Unclear	LH, FSH, E_2_, T, OW	BL23, CV6, PC6, ST36, SP6, EX-CA1	(34)
Kuang 2020	SD	PCOS	DHEA	EA	25	Interestrus	LH, FSH	CV6, SP6, EX-CA1	(35)
Yu 2019	SD	PCOS	Letrozole	MA	27	Unclear	LH, FSH, T, LH/FSH	CV4, ST36, SP6	(55)
Huang 2020	SD	PCOS	Letrozole	EA	14	Unclear	FSH, T, AMH	LR3, SP6	(36)
Chen 2017	SD	PCOS	TP	EA	35	Unclear	LH, FSH, T, LH/FSH	CV4, CV3, CV2, GV4, GV3, GV20	(37)
Zhang 2008	SD	PCOS	Letrozole	MA	15	Unclear	LH, FSH, E_2_, T, OW	CV3, CV4, SP6, EX-CA1	(38)
Zhang 2009	SD	PCOS	DHEA	MA	5	Unclear	LH, FSH, E_2_, T	CV3, CV4, SP6, EX-CA1	(39)
Sun 2013	SD	PCOS	Letrozole	EA	14	Unclear	E_2_, T	CV4, CV3	(40)
Yin 2019	SD	PCOS	Letrozole	EA	14	Unclear	AMH	CV4, CV3	(41)
Yuan 2021	SD	PCOS	Letrozole	MA	20	Interestrus	LH, FSH, T, OW	CV3, CV6, SP6, EX-CA1, ST10, ST40, BL23, BL20, BL18	(42)
Chen 2021	SD	PCOS	Levonorgestrel	MA	11	Unclear	LH, FSH, E_2_, T, AMH	CV4, CV3, CV6, SP6, EX-CA1	(43)
Peng 2020	SD	PCOS	DHEA	EA	17	Unclear	LH, FSH, T, LH/FSH	ST29, SP6	(44)
Zhao 2023	SD	PCOS	Letrozole	EA	14	Interestrus	T, FSH, E_2_, OW	CV4	(45)
Li 2016	SD	PCOS	DHEA	EA	25	Unclear	LH, FSH	SP6, EX-CA1, CV3, CV4	(11)
Yu 2005	SD	PMS	Senescence	EA	7	Unclear	E_2_, T	HT7, SP6, CV4	(46)
Ma 2008	SD	PMS	Senescence	MA	7	Unclear	E_2_	BL23, SP6, ST36	(47)
Liu 2003	SD	PMS	Senescence	EA	28	Unclear	LH, FSH, E_2_, NF	SP6, ST36, LR3	(48)
Kong 2016	SD	POF	Diethylstilbestrol	MA	30	Unclear	LH, FSH, E_2_	SP6, CV4, LR3, KI3	(49)
Chen 2020	SD	POF	VCD	MA	12	Unclear	LH, FSH, E_2_	SP6, CV4, ST36, DU20, BL32	(50)
Zhang 2015	SD	POF	CTX	MA	56	Unclear	LH, FSH, E_2_	CV4, CV6, ST29, LI4, SP4, LR3, SP6	(51)
Wang 2021	SD	POF	CTX	MA	10	Unclear	LH, FSH, E_2_	CV4, SP6, ST36, BL20, BL23, EX-CA1	(58)
Wang 2019	SD	POF	CTX	MA	21	Unclear	FSH, E_2_	DU20, CV4, EX-CA1, LI4, LR3, SP6	(58)
Ma 2022	SD	POI	CTX	MA	14	Unclear	LH, FSH, E_2_	BL54 through ST38	(52)
Wang 2018	SD	POI	VCD	EA	15	Interestrus	LH, FSH, E_2_, OW	BL33, ST25	(53)
Yan 2023	SD	POI	CTX	MA	14	Unclear	LH, FSH, E_2_	BL54 through ST38	(54)

SD, Sprague- Dawley; EA, Electroacupuncture; MA, Manual acupuncture; PCOS, Polycystic ovary syndrome; POF, Premature ovarian failure; POI, Premature ovarian insufficiency; PMS, Perimenopausal syndrome; OW, Ovarian weight; TP, Testosterone Propionate; DHEA, Dehydroepiandrosterone; CAS, 4-Vinyl-1cyclohexene; SPS; Sodium Prasterone Sulfate; PMSG, Pregnant Mare Serum Gonadotropin; VCD, 4-vinylcyclonhexenediepoxide; CTX, Cyclophosphamide.

### 3.3 Quality assessment

A comprehensive evaluation of 21 items was conducted in accordance with the ARRIVE guidelines. Based on the quality score-to-maximum score ratio, items 18, 19, and 20 were deemed suboptimal. Meanwhile, items 2, 3, 4, 6, 7, 9, 10, 12, 14, 16, 17, and 21 were classified as having an average rating, while items 1, 5, 8, 9, 11, 13, and 15 demonstrated an excellent level of adherence. The final aggregate score, calculated as 0.65, was considered to be of an average quality. A detailed breakdown of the quality assessment for the identified studies is presented in Table 2.

**Table 2:** Quality assessment according to the Animal Research Reporting *In Vivo* Experiment (ARRIVE) guidelines.

**Study**	**1**	**2**	**3**	**4**	**5**	**6**	**7**	**8**	**9**	**10**	**11**	**12**	**13**	**14**	**15**	**16**	**17**	**18**	**19**	**20**	**21**	**Total**	**References**
Xu 2019	2	1	2	1	1	1	2	2	3	1	1	1	1	1	1	1	1	0	0	0	1	24	(30)
Xu 2018	2	1	2	1	1	1	2	2	3	1	1	1	1	1	1	1	1	0	0	0	1	24	(31)
Sun 2016	2	1	2	1	1	1	1	2	3	1	1	1	1	1	1	2	1	0	0	0	1	24	(32)
Shen 2021	2	1	2	1	1	1	2	2	3	1	1	2	1	1	1	2	1	0	0	0	1	26	(33)
Li 2012	2	1	3	1	1	1	1	2	3	1	1	1	1	0	1	1	1	0	0	0	1	23	(34)
Kuang 2020	2	1	3	1	1	1	2	2	3	1	1	2	1	0	1	2	1	1	0	0	1	27	(35)
Yu 2019	2	1	2	2	1	1	2	2	3	1	1	1	1	1	1	2	1	0	0	0	1	26	(55)
Huang 2020	2	1	3	1	1	1	2	2	3	1	1	1	1	1	1	2	1	0	0	0	1	26	(36)
Chen 2017	2	1	2	1	1	1	1	2	3	1	1	1	1	1	1	1	1	0	0	0	1	23	(37)
Zhang 2008	2	1	2	1	1	1	1	2	3	1	1	2	1	0	1	1	1	0	0	0	1	23	(38)
Zhang 2009	2	1	2	1	1	1	1	2	3	1	1	2	1	0	1	1	1	0	0	0	1	23	(39)
Sun 2013	2	1	2	1	1	1	1	1	3	1	1	2	1	1	1	1	2	0	1	0	1	25	(40)
Yin 2019	2	1	2	1	1	1	1	2	3	1	1	2	1	1	1	2	2	1	1	0	1	28	(41)
Yuan 2021	2	1	2	1	1	1	1	2	3	1	1	2	1	0	1	2	1	0	0	0	1	24	(42)
Chen 2021	2	1	2	1	1	1	2	2	3	1	1	2	1	1	1	1	1	0	1	0	1	26	(43)
Peng 2020	2	1	2	1	1	1	1	2	3	1	1	2	1	1	1	2	1	0	1	0	2	27	(44)
Zhao 2023	2	1	2	1	1	1	1	2	3	1	1	2	1	1	1	3	1	0	1	0	1	27	(45)
Li 2016	2	1	2	1	1	1	1	2	3	1	1	2	1	1	1	2	1	0	0	0	1	25	(11)
Yu 2005	2	1	2	1	1	1	1	2	3	1	1	1	1	0	1	1	1	0	0	0	1	22	(46)
Ma 2008	2	1	2	1	1	1	1	2	3	1	1	1	1	0	1	1	2	0	0	0	1	23	(47)
Liu 2003	2	1	2	1	1	1	1	2	3	1	1	1	1	0	1	1	1	0	0	0	1	22	(48)
Kong 2016	2	1	2	1	1	1	1	2	3	1	1	1	1	0	1	1	1	0	0	0	1	22	(49)
Chen 2020	2	1	2	1	1	1	2	1	3	1	1	2	1	0	1	2	1	0	0	0	1	24	(49)
Zhang 2015	2	1	2	1	1	1	1	1	3	1	1	2	1	0	1	2	1	0	0	0	1	23	(51)
Wang 2021	2	1	2	1	1	1	2	1	3	1	1	1	1	0	1	1	2	0	0	0	1	23	(58)
Wang 2019	2	1	2	1	1	1	2	2	3	1	1	1	1	1	1	2	1	0	1	0	2	27	(59)
Ma 2022	2	1	2	1	1	1	1	2	3	1	1	1	1	0	1	2	1	0	0	0	1	23	(52)
Wang 2018	2	1	2	1	1	1	2	2	3	1	1	1	1	0	1	3	1	0	0	0	1	25	(53)
Yan 2023	2	1	2	1	1	1	2	2	3	1	1	1	1	1	1	2	1	0	1	0	2	27	(54)
Category score (quality obtained)	58	29	61	30	29	29	41	54	87	29	29	42	29	15	29	47	33	2	7	0	32	712	
Maximum score (quality expected)	58	58	87	58	29	58	58	58	116	58	29	58	29	29	29	87	58	29	29	29	58	1,102	
Ratio: quality score/maximum score	1	0.5	0.7	0.52	1	0.5	0.71	0.93	0.75	0.5	1	0.72	1	0.52	1	0.54	0.57	0.07	0.24	0	0.55	0.65	

(1). Study design; (2). Sample size; (3). Inclusion and exclusion criteria; (4). Randomization; (5). Blinding; (6). Outcome measures; (7). Statistical methods; (8). Experimental animal; (9). Experimental procedures; (10). Results; (11). Abstract; (12). Background; (13). Objectives; (14). Ethical statement; (15). Housing and husbandry; (16). Animal care and monitoring; (17). Interpretation/ scientific implications; (18). Generalis ability/ translation; (19). Protocol registration; (20). Data access; (21). Declaration of interests. Total: represents total score obtained by each study out of a maximum of 38 point.

### 3.4 Risk of bias

The quality assessment was conducted using the SYRCLE risk assessment tool for animal experiments. The selected studies comprised seven with a low risk of sequence generation (30, 31, 42, 47, 50, 53, 55), eight with a low risk in baseline characteristics (35, 36, 43, 44, 52–55), and three with a low risk of random results (34, 37, 39). Additionally, all 29 studies exhibited a low risk in terms of incomplete result data, selective result reporting, and bias from other sources. A comprehensive overview of the risk of bias is provided in Table 3.

**Table 3:** Analysis of the risk of bias, based on SYRCLES methodology.

**Studies**	**1**	**2**	**3**	**4**	**5**	**6**	**7**	**8**	**9**	**10**	**References**
Xu 2019	y	?	n	?	n	n	n	y	y	y	(30)
Xu 2018	y	?	n	?	n	n	n	y	y	y	(31)
Sun 2013	?	?	n	?	n	n	n	y	y	y	(32)
Shen 2021	?	?	n	?	n	n	n	y	y	y	(33)
Li 2012	?	?	n	?	n	y	n	y	y	y	(34)
Kuang 2020	?	y	n	?	n	n	n	y	y	y	(35)
Yu 2019	y	y	n	?	n	n	n	y	y	y	(55)
Huang 2020	?	y	n	?	n	n	n	y	y	y	(36)
Chen 2017	?	?	n	?	n	y	n	y	y	y	(37)
Zhang 2008	?	?	n	?	n	n	n	y	y	y	(38)
Zhang 2009	?	?	n	?	n	y	n	y	y	y	(39)
Sun 2013	?	?	n	?	n	n	n	y	y	y	(40)
Yin 2019	?	?	n	?	n	n	n	y	y	y	(41)
Yuan 2021	y	?	n	?	n	n	n	y	y	y	(42)
Chen 2021	?	y	n	?	n	n	n	y	y	y	(43)
Peng 2020	?	y	n	?	n	n	n	y	y	y	(44)
Zhao 2023	?	?	n	?	n	n	n	y	y	y	(45)
Li 2016	?	?	n	?	n	n	n	y	y	y	(11)
Yu 2005	?	?	n	?	n	n	n	y	y	y	(46)
Ma 2008	y	?	n	?	n	n	n	y	y	y	(47)
Liu 2003	?	?	n	?	n	n	n	y	y	y	(48)
Kong 2016	?	?	n	?	n	n	n	y	y	y	(49)
Chen 2020	y	?	n	?	n	n	n	y	y	y	(50)
Zhang 2015	?	?	n	?	n	n	n	y	y	y	(51)
Wang 2021	?	?	n	?	n	n	n	y	y	y	(58)
Wang 2019	?	?	n	?	n	n	n	y	y	y	(59)
Ma 2022	?	y	n	?	n	n	n	y	y	y	(52)
Wang 2018	y	y	n	?	n	n	n	y	y	y	(53)
Yan 2023	y	y	n	?	n	n	n	y	y	y	(54)

1. Sequence generation; 2. Baseline characteristics; 3. Allocation concealment; 4. Random housing; 5. Blinding of participants and personnel; 6. Random outcome assessment; 7. Blinding of outcome assessment; 8. Incomplete outcome data; 9. Selective outcome reporting; 10. Other bias; y: low risk of bias; ?: unclear; n: high risk of bias.

### 3.5 Effect of acupuncture or electroacupuncture on ovarian function

#### 3.5.1 Polycystic ovarian syndrome

**Figure 2:**
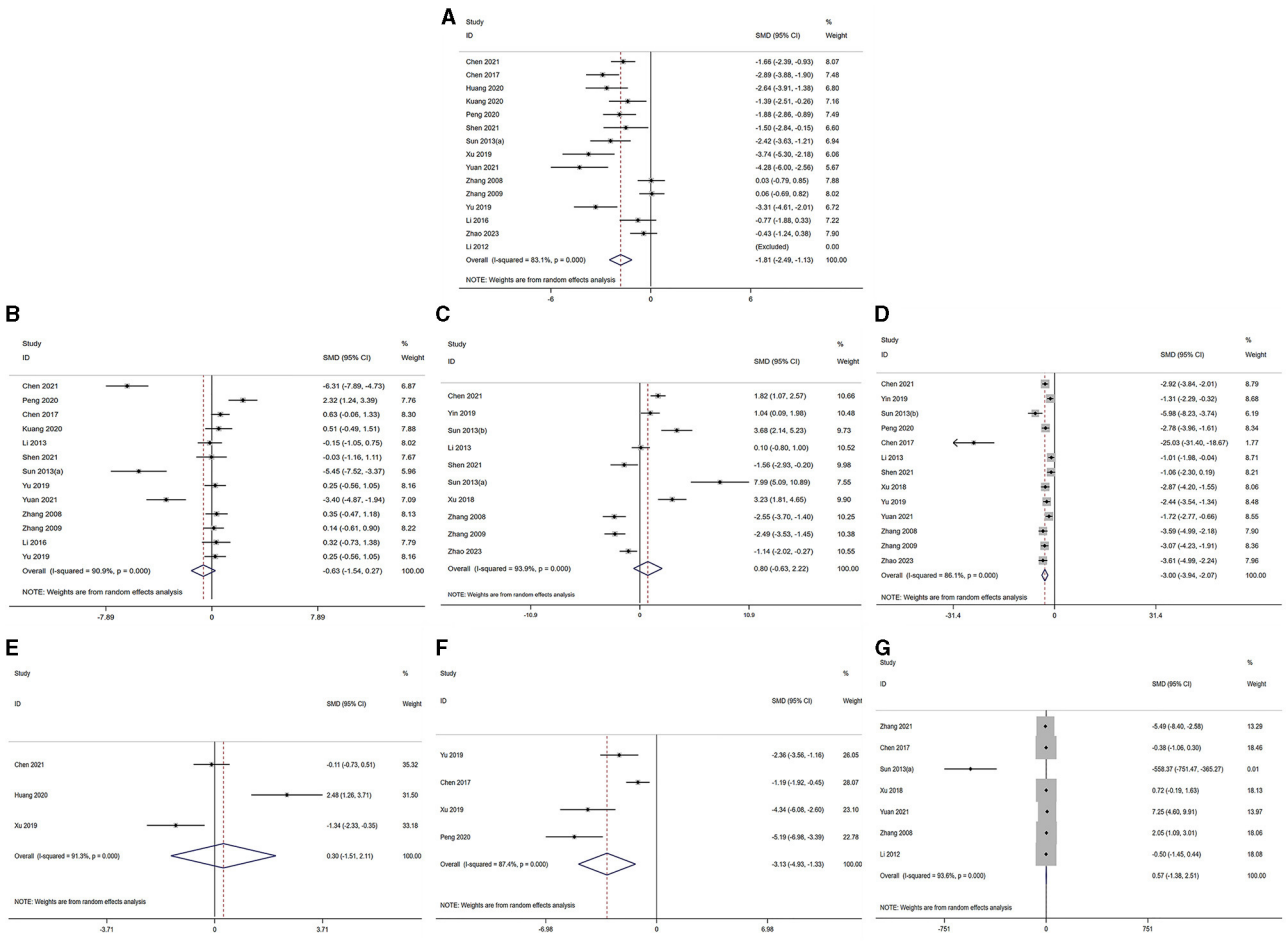
Forest plot of comparison acupuncture vs. control in model of PCOS. The statistical method is random effect. **(A)** Forest map of LH; **(B)** Forest map of FSH; **(C)** Forest map of E_2_; **(D)** Forest map of T; **(E)** Forest map of AMH; **(F)** Forest map of LH/FSH; **(G)** Forest map of OW. LH, luteinizing hormone; FSH, follicle-stimulating hormone; T, testosterone; E_2_, estradiol; AMH, Anti-Müllerian hormone; OW, Ovarian weight.

The serum levels of LH, FSH, T, E_2_, LH/FSH ratio and AMH were subjected to analysis across the selected studies (30–40, 42–45, 55–57). The findings indicated the following: in comparison to the model group, the LH level demonstrated a significant decrease in the acupuncture group [acupuncture group *n* = 191, model group *n* = 190, *I*^2^ = 84.40%, SMD = −1.90 (95% CI −2.60 to −1.19); Z = 10.76, *P* = 0.000]. Similarly, the T level exhibited a significant reduction in the acupuncture group [acupuncture group *n* = 154, model group *n* = 153, *I*^2^ = 86.94%, SMD = −3.14 (95% CI −4.10 to −2.18); Z = 14.77, *P* = 0.023], and the ratio also showed a significant decrease in the acupuncture group [acupuncture group *n* = 49, model group *n* = 49, *I*^2^ = 86.94%, SMD = −3.13 (95% CI −4.93 to −1.33); Z = 3.41, *P* = 0.001]. Conversely, the E_2_ level [acupuncture group *n* = 153, model group *n* = 152, *I*^2^ = 94.47%, SMD = 0.85 (95% CI −0.64 to 2.33); Z = 0.88, *P* = 0.40], the FSH level [acupuncture group *n* = 149, model group *n* = 148, *I*^2^ = 91.59%, SMD = −0.67 (95% CI −1.60 to 0.27); Z = −1.05, *P* = 0.31], and the AMH level [acupuncture group *n* = 40, model group *n* = 40, *I*^2^ = 92.06%, SMD = 0.32 (95% CI −1.57 to 2.21); Z = 0.28, *P* = 0.80] did not exhibit a significant difference in the acupuncture group. The corresponding forest plots for each outcome measurement are illustrated in Figures 2A–F.

The analysis of ovarian weight, as reported in studies (31, 32, 34, 37, 38, 42, 45), revealed that in the acupuncture group, there was no significant difference in ovarian weight [acupuncture group *n* = 82, model group *n* = 81, *I*^2^ = 91.6%, SMD = 0.76 (95% CI −1.25 to 2.77); Z = 0.21, *P* = 0.838] when compared to the model group. Further details pertaining to the ovarian morphology in PCOS are presented in Figure 2G.

#### 3.5.2 Decreased ovarian function

**Figure 3:**
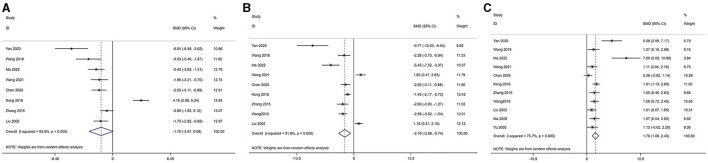
Forest plot of comparison: acupuncture vs. control in model of decreased ovarian function. The statistical method is random effect. **(A)** Forest map of LH; **(B)** Forest map of FSH; **(C)** Forest map of E_2_. LH, luteinizing hormone; FSH, follicle-stimulating hormone; T, testosterone; E_2_, estradiol.

Based on the disease characteristics, we classified POI, POF, and PMS into the category of decreased ovarian function. The serum levels of LH, FSH, and E_2_ were analyzed across studies (46, 47, 49–54, 58–60). The outcomes are as follows: Serum E_2_ level [acupuncture group *n* = 113, model group *n* = 113, *I*^2^ = 78.00%, SMD = 1.87 (95% CI 1.16–2.58); Z = 3.33, *P* = 0.008] demonstrated a significant increase compared to the model group. LH level [acupuncture group *n* = 82, model group *n* = 82, *I*^2^ = 94.14%, SMD = −1.81 (95% CI −3.65 to 0.02); Z = −1.75, *P* = 0.124] and FSH (acupuncture group *n* = 96, model group *n* = 96, *I*^2^ = 92.61%, SMD = −2.30 [95% CI −3.78 to 0.81]; Z = −2.19, *P* = 0.06) in the acupuncture group were not significantly different from the model group. These findings are visually represented in Figure 3.

## 4 Heterogeneity and bias analysis

### 4.1 Heterogeneity analysis

**Figure 4:**
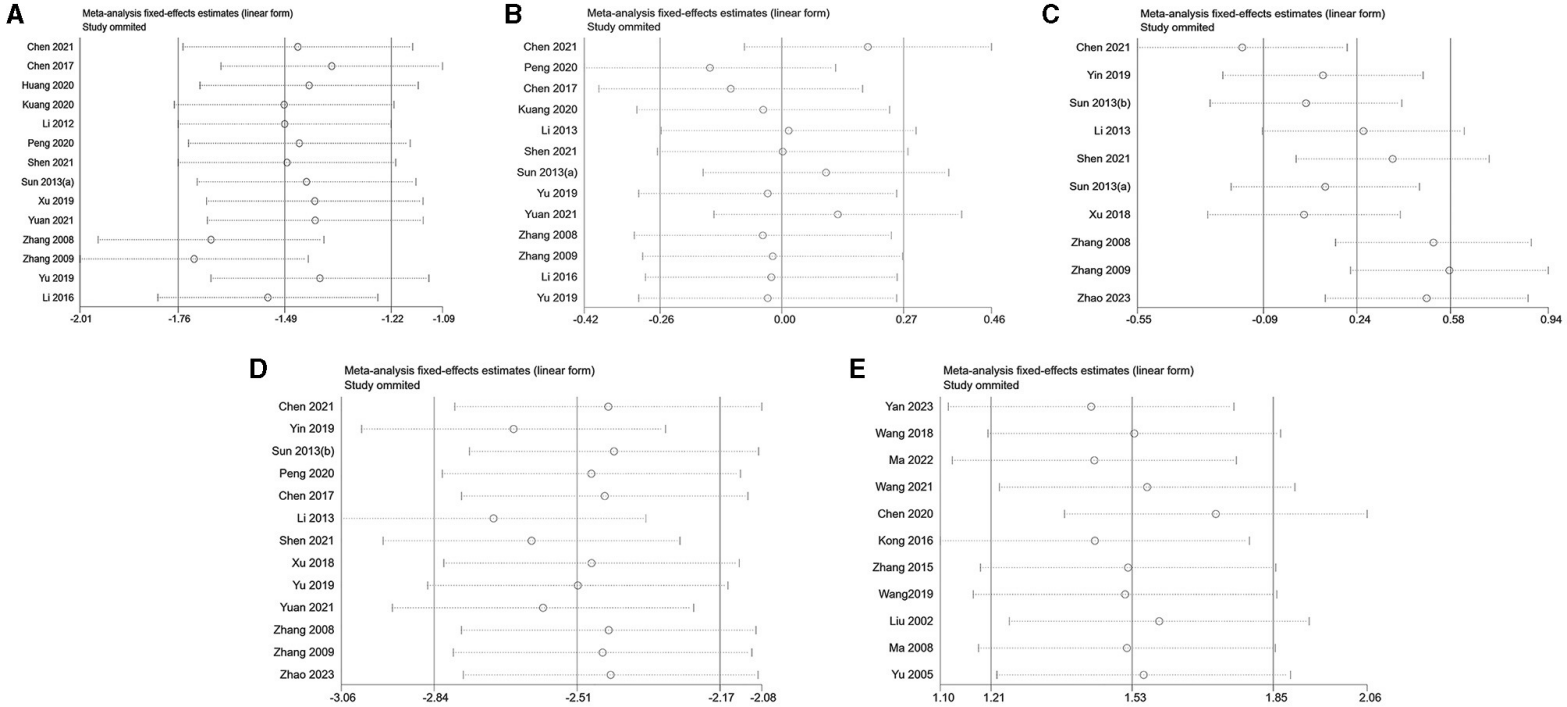
Sensitivity analysis. **(A)** LH of PCOS; **(B)** FSH of PCOS; **(C)** E_2_ of PCOS; **(D)** T of PCOS; **(E)** E_2_ of decreased ovarian function.

In this study, a sensitivity analysis was conducted for each outcome index that included more than 10 pieces of literature. The analysis encompassed 15 articles on LH analysis, 13 articles on FSH analysis, 10 articles on E_2_ analysis, 13 articles on T analysis in PCOS, and 11 articles on E_2_ analysis in decreased ovarian function (Figure 4). Remarkably, the omission of Chen's study in 2021 (43) significantly influenced the stability of results in the E_2_ analysis within the context of PCOS. This impact could potentially be attributed to variations in the modeling drugs employed in this particular study, introducing heterogeneity in the outcomes. Furthermore, the heterogeneity of other results persisted, as evidenced by the continued detection using Egger's test.

### 4.2 Publication bias analysis

Egger's test was employed to examine the bias within the included literature, encompassing 15 types of literature in LH analysis, 13 types of literature in FSH analysis, 13 types of literature in T analysis in PCOS, and 11 articles on E_2_ analysis in decreased ovarian function. The findings reveal that a *P* < 0.05 for LH and E_2_ indicates the presence of publication bias, necessitating supplementary correction methods such as the trim-and-fill technique. Conversely, the analysis of FSH and T indicated a relatively low likelihood of publication bias, as summarized in Table 4.

**Table 4:** Egger's test.

**Name**	***t***	***P* > |t|**	**95% CI**	
**PCOS**				
LH	−3.84	0.00	−11.57	−3.20
FSH	−3.54	0.01	−15.41	−1.71
T	−5.75	0.00	−14.88	−3.47
**Decreased ovarian function**				
E_2_	3.68	0.01	2.15	9.04

### 4.3 Trim and filling method

**Figure 5:**
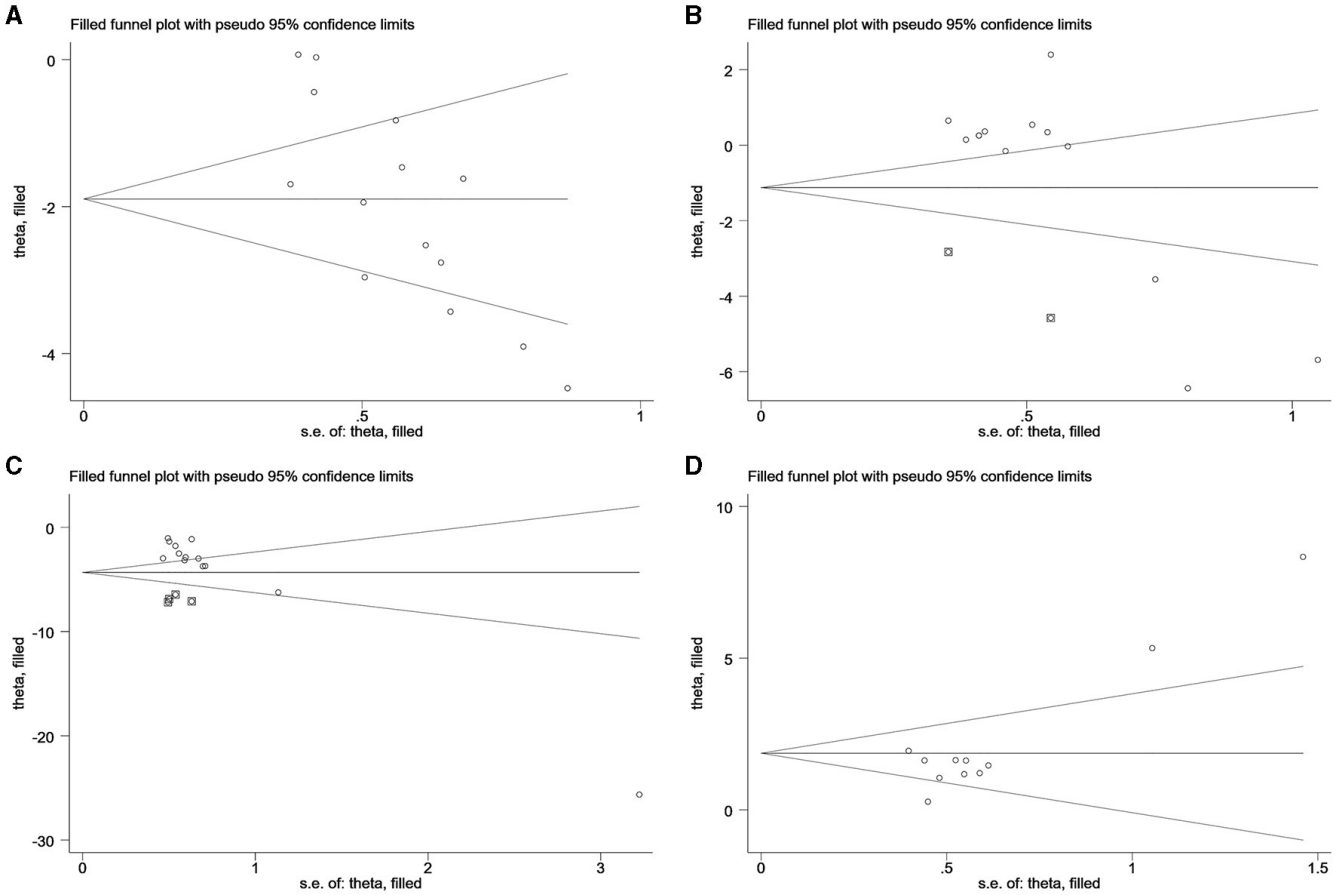
Trim and filling method. **(A)** LH of PCOS; **(B)** FSH of PCOS; **(C)** T of PCOS; **(D)** E_2_ of decreased ovarian function.

In Figure 5, no additional data for LH, FSH, T in PCOS, and E_2_ in decreased ovarian function are apparent. The analysis indicates that the data for LH in PCOS and E_2_ in decreased ovarian function have undergone no filling study, suggesting the stability of the results.

For LH analysis, two rounds of LINEAR iterations were performed with no additional articles identified, resulting in *Q* = 83.59, *P* = 0.00 < 0.05. This signifies the stability of the original results. Subsequently, a random-effects model was employed, yielding a combined result of IOR = 0.15 for the effect indicators, and a 95% CI of (0.07–0.30).

Similarly, the analysis of E_2_ underwent two rounds of LINEAR iterations with no additional articles added, resulting in *Q* = 45.56, *P* = 0.00 < 0.05, indicating the stability of the original results. The subsequent use of a random-effects model produced a combined effect indicator of IOR = 6.47, with a 95% CI of (3.18–13.16).

Notably, two points in the FSH plot and four points in the T plot, indicated as squares, are suggested as potential effect sizes for future inclusion. Combining the above forest plots ensures the symmetry of the funnel plots, eliminating publication bias by consistently incorporating articles with similar results from corresponding studies expressed in the plots.

## 5 Discussion

In this comprehensive meta-analysis, our objective was to address the effectiveness of acupuncture in ameliorating ovarian dysfunction, drawing insights from the collective data of 623 rats across 29 articles. The study outcomes revealed that acupuncture led to a noteworthy reduction in serum LH, T and LF/FSH ratio levels in rats modeled with PCOS. Additionally, acupuncture exhibited a significant increase in serum E_2_ levels in rats modeled with decreased ovarian function. Intriguingly, despite these observed effects, acupuncture did not yield a significant alteration in ovarian volume in the context of PCOS.

Absolutely, our statement provides a concise and accurate summary of the crucial roles played by LH and FSH in ovarian function. These hormones, secreted by the pituitary gland, are instrumental in orchestrating the complex processes involved in the development of follicles and oocytes within the ovaries. FSH is essential for promoting the maturation of follicles, working in tandem with LH to facilitate ovulation. LH, in turn, acts synergistically with FSH, particularly during the late follicular stage, to promote the development and optimal functioning of follicles. The regulation of the hypothalamus-pituitary-ovary axis (HPOA) by LH and FSH is a key aspect of maintaining ovarian function. This regulatory mechanism plays a central role in promoting follicular development and finely tuning the secretion of estrogen, thereby contributing significantly to the overall regulation of the female reproductive system (61). Our statement aptly captures the interconnected nature of sex hormone regulation and how acupuncture can impact these dynamics. T serves as a crucial precursor in the biosynthesis of E_2_ and exerts a negative feedback regulatory effect on the hypothalamus-pituitary axis (62, 63). LH plays a significant role in the regulation of T synthesis, and therefore, the effects of acupuncture on T align with its impact on LH. For PCOS, characterized by increased LH secretion, elevated LH/FSH ratio, and elevated T levels in some patients, research findings indicate that both acupuncture and electroacupuncture can significantly reduce serum LH, T, and LH/FSH levels. This suggests that acupuncture holds promise in effectively addressing sex hormone level disorders in PCOS and potentially restoring abnormal ovarian function. E_2_ is a key component in follicular development and maturation, exerts both positive and negative feedback effects on the hypothalamus (64, 65). Additionally, E_2_ plays a decisive role in maintaining ovarian function and mitigating ovarian aging (66). This highlights the potential of acupuncture to influence a range of sex hormones, contributing to the restoration of hormonal balance and, consequently, improved ovarian function. The study outcomes reveal that both acupuncture and electroacupuncture interventions have the potential to enhance serum E_2_ levels across various degrees of ovarian hypoplasia, thereby contributing to the alleviation of the ovarian hypoplasia process. However, the observation of no significant difference in ovarian weight between the acupuncture group and the model group in PCOS suggests the need for cautious interpretation. This lack of significance may be associated with the relatively short duration of acupuncture treatment in animal experiments and the limited number of articles included in this study (67). It underscores the importance of considering these factors and highlights the necessity of supplementing relevant outcome indicators in future studies to provide a more comprehensive understanding of the impact of acupuncture on ovarian weight in PCOS models.

In the current study, it was observed that only six of the included articles proposed the collection of serum samples during the inter-estrous period in model animals (30, 31, 35, 42, 45, 53). However, a sensitivity analysis was conducted to assess the impact of excluding any of the articles that sampled during the interestrus period from LH, FSH, and T analyses. Remarkably, the results indicated that such exclusions had no significant effect on the overall meta-analysis results. Given the similarity between the animal estrous cycle and the human menstrual period, it is noteworthy that there are substantial changes in serum sex hormones across different estrous cycles (68). This factor may exert an influence on the outcomes of our study (69). Follicular development exhibits a close association with the level of E_2_, especially in patients with PCOS during the anogenital follicular phase. Here, an increase in E_2_ may play a role in suppressing oocyte meiosis, potentially resulting in abnormal oocyte maturation (70). In the context of the early stages of PMS, a reduction in LH and T levels indicates a decline in ovarian function. It is crucial to recognize that hormonal variations at different points in time can have diverse effects on ovarian function and follicular development. Therefore, when investigating alterations in sex hormones, the changes in the motility cycle emerge as a significant factor that cannot be overlooked.

This meta-analysis puts forth the proposition that acupuncture exhibits the capacity to reduce elevated hormones, elevate lowered hormones, and normalize disordered hormone levels in the treatment of dysfunctional ovarian diseases. Hence, our inference is that the regulation of ovarian dysfunction through acupuncture or electroacupuncture primarily occurs via the modulation of serum sex hormones.

**Figure 6:**
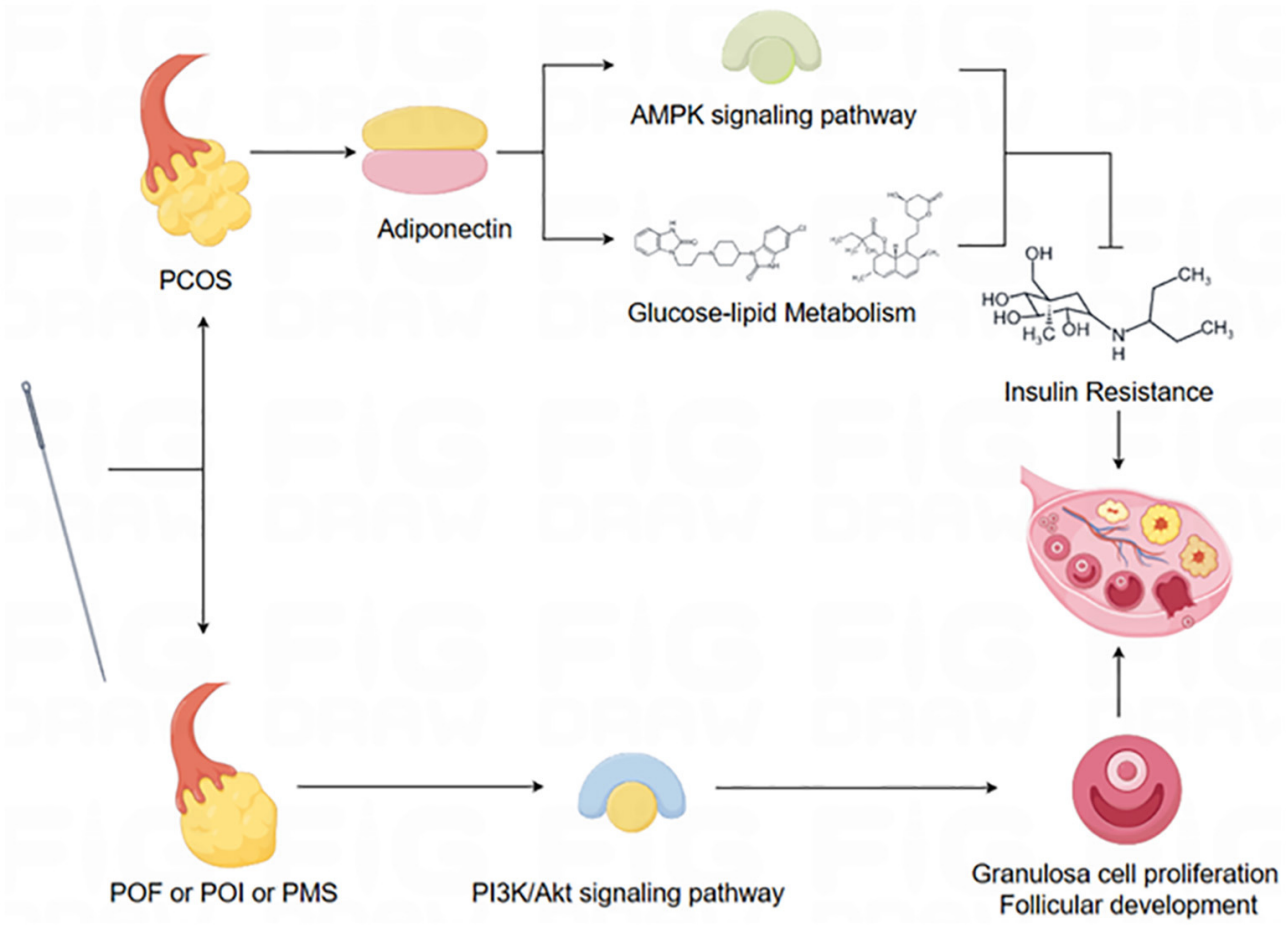
Schematic diagram of the mechanism of action of acupuncture.

Additionally, previous studies have elucidated the multifaceted impact of acupuncture or electroacupuncture on ovarian function. These effects encompass diverse mechanisms, such as modulating protein content within ovarian tissue, altering sex hormone receptor activity, modifying gene expression associated with reproductive factors, influencing metabolic factors, and modulating various signaling pathways (41, 48, 71–78), and alteration of the expression of various proteins in the hypothalamus (79, 80). For example, most PCOS patients are usually accompanied by insulin resistance (IR), and lipocalin can increase insulin sensitivity and promote glucose metabolism, which is a key signaling factor in the regulation of glucose-lipid metabolism, and plays an important role in regulating the modulation of reproductive and metabolic disorders (81–85). Studies have demonstrated that acupuncture can ameliorate glycolipid metabolism in PCOS by downregulating lipocalin receptor expression, upregulating lipocalin protein expression, and activating the AMP-activated protein kinase (AMPK) pathway (86–88). POF, POI, and PMS are characterized by reductions in ovarian volume and cell numbers. However, acupuncture has been shown to increase granulosa cell and follicle numbers by activating the Phosphoinositide 3-kinase/Protein Kinase B (PI3K/AKT) signaling pathway, thereby improving ovarian function and fertility (59, 89). In conclusion, the confirmed regulatory effect of acupuncture or electroacupuncture on ovarian function provides a novel therapeutic perspective for the treatment of diseases associated with ovarian function (Figure 6).

## 6 Strength and limitations

The study did not undertake a comprehensive analysis of all indices. Given the inclusion of diverse diseases and the inconsistent changes observed in serum sex hormones and ovarian morphology across these conditions, the effects of each hormone were individually accounted for. This approach allowed for a nuanced explanation of hormonal changes across different diseases.

A notable portion of the articles did not account for the impact of different estrous cycles on outcomes during serum sample extraction. Considering the relevance of estrous cycle recovery in animal models as an important indicator of acupuncture response in diseases related to ovarian dysfunction, future studies should meticulously consider the fluctuation of sex hormone levels across different estrous cycles. Researchers are advised to choose outcome indicators judiciously to more accurately reflect acupuncture efficacy.

The reliance on specific outcome indicators to gauge the integrative modulatory effects of acupuncture on disorders of abnormal ovarian function may potentially constrain the comprehensive interpretation of acupuncture efficacy. And since changes in serum sex hormones are a common indicator for assessing the efficacy of acupuncture in clinical trials, we likewise chose serum sex hormones as an observational indicator of efficacy in our study. However, in future research endeavors, we should take full advantage of the opportunity provided by animal experiments to incorporate additional outcome indicators. This approach would better summarize the multifaceted effects of acupuncture on ovarian function. For example, parameters such as protein content, gene expression and signaling pathway alterations must be studied in depth.

The predominance of studies focused on PCOS in this analysis may restrict the generalizability of the findings to other ovarian dysfunction diseases. Nevertheless, it is essential to highlight that this study marks the first endeavor to evaluate the effects of acupuncture or electroacupuncture on improving animal models of ovarian dysfunction across various diseases, providing insights into the underlying mechanisms of acupuncture or electroacupuncture. The aim of this study was to comprehensively observe the efficacy of acupuncture therapy on ovarian dysfunction diseases, and the previous analysis of acupuncture and electroacupuncture by subgroups did not appear to have a significant impact on the analytical results, so this study did not describe the subgroup analysis of acupuncture and electroacupuncture. And due to the limited number of articles we screened, especially POF, PMS and POI, subgroup analysis of acupuncture and electroacupuncture was difficult to achieve, so future studies should further include more articles to further analyze and analyze the efficacy of acupuncture and electroacupuncture.

Finally, this study observed the effects of acupuncture on various ovarian dysfunctions through animal experiments. In future research, we will focus on observing whether acupuncture has similar therapeutic effects in clinical studies, which will require further in-depth analysis and exploration.

## 7 Conclusion

This meta-analysis underscores that acupuncture treatment has the potential to enhance ovarian function in PCOS, POF, POI, and PMS model rats by effectively regulating serum sex hormones. The findings of this review provide a valuable foundation for future research into the therapeutic effects of acupuncture or electroacupuncture. It is anticipated that this comprehensive review will serve as a noteworthy reference in this evolving field of research, encouraging further exploration and insights into the application of acupuncture in the context of ovarian dysfunction.

## Data availability statement

The original contributions presented in the study are included in the article/supplementary material, further inquiries can be directed to the corresponding authors.

## Author contributions

YZ: Writing – review & editing, Writing – original draft. YL: Writing – review & editing. LL: Writing – review & editing. JH: Writing – review & editing. HW: Writing – review & editing. LJ: Writing – review & editing.
