# Supplementary material for: Efficacy of acupuncture in animal models of various ovarian dysfunctions: a systematic review and meta-analysis
Source: Front Med (Lausanne). 2024 Jun 20;11:1348884. doi: 10.3389/fmed.2024.1348884 (PMC11222413; doi:10.3389/fmed.2024.1348884)
Supplement: Supplementary file 1 [file Data_Sheet_1.docx]

**1. Chinese National Knowledge Infrastructure (CNKI)**

**#1**= subject%='PCOS' or title%=' PCOS'

**#2=** subject%='ovarian hyperstimulation' or title%='ovarian hyperstimulation'

**#3**= subject%='PCOS' or title%=' PCOS'

**#4**= subject%='premature ovarian failure' or title%='premature ovarian failure'

**#5**= subject%='ovarian reserve function' or title%='ovarian reserve function'

**#6**= Subject%='low ovarian response' or title%='low ovarian response'

**#7**= Subject%='perimenopausal syndrome' or title%='perimenopausal syndrome'

**#8**= Subject%='menopausal syndrome' or title%='menopausal syndrome'

**#9**= **#1** OR **#2** OR **#3** OR **#4** OR **#5** OR **#6** OR **#7** OR **#8**

**#10**= Subject%='acupuncture' or title%='acupuncture'

**#11**= Subject%='electro acupuncture' or title%='electro acupuncture'

**#12**= **#10** OR **#11**

**#13**= Subject%='rat' or title%='rat')

**#14**= Subject%='animal experiment' or Title%='Animal Experiment'

**#15**= subject%='animal' or title%='animal')

**#16**= subject%='animal model' or title%='animal model'

**#17**=**#13** OR **#14** OR **#15** OR **#16**

**#18**= **#9** AND **#12** AND **#17**

**2. Wanfang Data**

**#1**= Topic: (polycystic ovary syndrome or ovarian hyperstimulation or ovarian function or premature ovarian failure or ovarian reserve function or low ovarian response or perimenopausal syndrome or menopausal syndrome)

**#2**= Topic: (acupuncture or electroacupuncture)

**#3**= Topic: (rats or animal experiments or animals or animal models)

**#4= #1** AND **#2** AND **#3**

**3. VIP information database**

**#1**= (title or keyword=polycystic ovary syndrome

**#2**= title or keyword=ovarian hyperstimulation

**#3**= title or keyword=ovarian function

**#4**= title or keyword=premature ovarian failure

**#5**= title or keyword=ovarian reserve function

**#6**= title or keyword=ovarian hyporesponsiveness

**#7**= title or keyword=perimenopausal syndrome

**#8**= title or keyword=menopausal syndrome

**#9**=**#1** OR **#2** OR **#3** OR **#4** OR **#5** OR **#6** OR **#7** OR **#8**

**#10**= title or keyword=acupuncture

**#11**=title or keyword=electroacupuncture

**#12**=**#10** OR **#11**

**#13=** title or keyword=rat

**#14**= title or keyword=animal experiment

**#15**= title or keyword=animal

**#16**= title or keyword=animal model

**#17**= **#13** OR **#14** OR **#15** OR **#16**

**#18= #9** AND **#12** AND **#17**

**4. China Biology Medicine (CBM disc)**

**#1**= "Polycystic ovary syndrome" [Common field: intelligent]

**#2**= "ovarian hyperstimulation" [Common field: intelligent]

**#3**= "ovarian function" [Common field: intelligent] AND "or" [Common field: intelligent] AND "premature ovarian failure" [Common field: intelligent] AND "or" [Common field: intelligent] AND "ovarian reserve function" [Common field: intelligent]

**#4**= "ovarian hyporesponsiveness" [Common Fields: Intelligence] AND "or" [Common Fields: Intelligence] AND "Perimenopausal Syndrome" [Common Fields: Intelligence] AND "or" [Common Fields: Intelligence] AND "Climacteric Syndrome"

**#5**= **#1** OR **#2** OR **#3** OR **#4**

**#6**= "Acupuncture" [Common Fields: Intelligence]

**#7**= "Electroacupuncture" [Common Fields: Intelligence]

**#8**= **#6** OR **#7**

**#9**= "Rat" [Common Fields: Intelligence]

**#10**= "Animal Experiment" [Common Fields: Intelligence]

**#11**= Animal" [Common Field: Intelligence]

**#12**= Animal Model" [Common Field: Intelligence]

**#13**= **#9** OR **#10** OR **#11** OR **#12**

**#14**=**#5** AND **#8** AND **#13**

**5.Pubmed**

**#1**= "Polycystic Ovary Syndrome"[MeSH Terms]

**#2**= "ovary syndrome polycystic"[Title/Abstract] OR "syndrome polycystic ovary"[Title/Abstract] OR "stein leventhal syndrome"[Title/Abstract] OR "stein leventhal syndrome"[Title/Abstract] OR "syndrome stein leventhal"[Title/Abstract] OR "sclerocystic ovarian degeneration"[Title/Abstract] OR ((("Ovarian"[All Fields] OR "ovarians"[All Fields]) AND ("degenerate"[All Fields] OR "degenerated"[All Fields] OR "degenerately"[All Fields] OR "degenerates"[All Fields] OR "degenerating"[All Fields] OR "Degeneration"[All Fields] OR "degenerations"[All Fields])) AND "Sclerocystic"[Title/Abstract]) OR "sclerocystic ovary syndrome"[Title/Abstract] OR "polycystic ovarian syndrome"[Title/Abstract] OR "ovarian syndrome polycystic"[Title/Abstract] OR "polycystic ovary syndrome 1"[Title/Abstract]

**#3**= **#1** OR **#2**

**#4**= "Ovarian Hyperstimulation Syndrome"[MeSH Terms]

**#5**= "ovarian hyperstimulation syndrome"[MeSH Terms] OR ("Ovarian"[All Fields] AND "Hyperstimulation"[All Fields] AND "syndrome"[All Fields]) OR "ovarian hyperstimulation syndrome"[All Fields] OR ("Hyperstimulation"[All Fields] AND "syndrome"[All Fields] AND "Ovarian"[All Fields]) OR "hyperstimulation syndrome ovarian"[All Fields] OR ((("hyperstimulated"[All Fields] OR "Hyperstimulation"[All Fields] OR "hyperstimulations"[All Fields]) AND ("syndrom"[All Fields] OR "syndromal"[All Fields] OR "syndromally"[All Fields] OR "syndrome"[MeSH Terms] OR "syndrome"[All Fields] OR "Syndromes"[All Fields] OR "syndrome s"[All Fields] OR "syndromic"[All Fields] OR "syndroms"[All Fields])) AND "Ovarian"[Title/Abstract]) OR "ovarian hyperstimulation syndromes"[Title/Abstract]

**#6= #4 OR #5**

**#7=** (((((Test, Ovarian Function[Title/Abstract]) OR (Tests, Ovarian Function[Title/Abstract])) OR (Ovarian Function Test[Title/Abstract])) OR (Function Tests, Ovarian[Title/Abstract])) OR (Function Test, Ovarian[Title/Abstract])) OR (ovarian function[Title/Abstract])

**#8=** ((((((((((((Ovarian Insufficiency, Primary[Title/Abstract]) OR (Ovarian Failure, Premature[Title/Abstract])) OR (Premature Ovarian Failure[Title/Abstract])) OR (Gonadotropin-Resistant Ovary Syndrome[Title/Abstract])) OR (Gonadotropin Resistant Ovary Syndrome[Title/Abstract])) OR (Resistant Ovary Syndrome[Title/Abstract])) OR (Premature Menopause[Title/Abstract])) OR (Decreased ovarian reserve[Title/Abstract])) OR (Diseased ovarian reserve[Title/Abstract])) OR (amenorrhea[Title/Abstract])) OR (Premature Menopause[Title/Abstract])) OR (Decreased ovarian reserve[Title/Abstract])) OR (Diseased ovarian reserve[Title/Abstract])

**#9=** "Acupuncture"[Mesh]

**#10**= "electroacupuncture"[Title/Abstract]

**#11**= **#9** OR **#10**

**#12**= (Climacteric syndrome[Title/Abstract]) OR (Perimenopausal syndrome[Title/Abstract])

**#13**= **#3** OR **#6** OR **#7** OR **#8** OR **#11** OR **#12**

**#14**= "Rats"[Mesh]

**#15**= ((((((Rattus[Title/Abstract]) OR (Rattus norvegicus[Title/Abstract])) OR (Rats, Norway[Title/Abstract])) OR (Rats, Laboratory[Title/Abstract])) OR (Laboratory Rat[Title/Abstract])) OR (Laboratory Rats[Title/Abstract])) OR (Rat, Laboratory[Title/Abstract])

**#16**= **#14** OR **#15**

**#17**= "Models, Animal"[Mesh]

**#18**= ((((((((((((((Animal Model[Title/Abstract]) OR (Animal Models[Title/Abstract])) OR (Model, Animal[Title/Abstract])) OR (Laboratory Animal Models[Title/Abstract])) OR (Animal Model, Laboratory[Title/Abstract])) OR (Animal Models, Laboratory[Title/Abstract])) OR (Laboratory Animal Model[Title/Abstract])) OR (Model, Laboratory Animal[Title/Abstract])) OR (Models, Laboratory Animal[Title/Abstract])) OR (Experimental Animal Models[Title/Abstract])) OR (Animal Model, Experimental[Title/Abstract])) OR (Animal Models, Experimental[Title/Abstract])) OR (Experimental Animal Model[Title/Abstract])) OR (Model, Experimental Animal[Title/Abstract])) OR (Models, Experimental Animal[Title/Abstract])

**#19**= **#17** OR **#19**

**#20**= "Animal Experimentation"[Mesh]

**#21**= ((((((((((Experimentation, Animal[Title/Abstract]) OR (Animal Research[Title/Abstract])) OR (Research, Animal[Title/Abstract])) OR (Animal Experimental Use[Title/Abstract])) OR (Animal Experimental Uses[Title/Abstract])) OR (Experimental Use, Animal[Title/Abstract])) OR (Experimental Uses, Animal[Title/Abstract])) OR (Animal Experiments[Title/Abstract])) OR (Animal Experiment[Title/Abstract])) OR (Experiment, Animal[Title/Abstract])) OR (Experiments, Animal[Title/Abstract])

**#22**= **#20** OR **#21**

**#23**= "Animals"[Mesh]

**#24**= ((Animalia[Title/Abstract]) OR (Animal[Title/Abstract])) OR (Metazoa[Title/Abstract])

**#25**= **#23** OR **#24**

**#26= #16** OR **#19** OR **#22** OR **#25**

**#27= #13 AND #26**

**6.Embase**

**#1**='Polycystic Ovary Syndrome':ab,ti or 'Ovary Syndrome, Polycystic':ab,ti or 'Syndrome, Polycystic Ovary':ab,ti or 'Stein-Leventhal Syndrome':ab,ti or 'Stein Leventhal Syndrome':ab,ti or 'Syndrome, Stein-Leventhal':ab,ti or 'Sclerocystic Ovarian Degeneration':ab,ti or 'Ovarian Degeneration, Sclerocystic':ab,ti or 'Sclerocystic Ovary Syndrome':ab,ti or 'Polycystic Ovarian Syndrome':ab,ti or 'Ovarian Syndrome, Polycystic':ab,ti or 'Polycystic Ovary Syndrome 1':ab,ti or 'Sclerocystic Ovaries':ab,ti or 'Ovary, Sclerocystic':ab,ti or 'Sclerocystic Ovary':ab,ti or 'Ovarian hyperstimulation syndrome':ab,ti or 'Hyperstimulation Syndrome, Ovarian':ab,ti or 'Hyperstimulation Syndromes, Ovarian':ab,ti or 'Ovarian Hyperstimulation Syndromes':ab,ti or 'Ovarian Hyperstimulation Syndrome, Familial Gestational Spontaneous':ab,ti or 'Ovarian Insufficiency, Primary':ab,ti or 'Ovarian Failure, Premature':ab,ti or 'Premature Ovarian Failure':ab,ti or 'Gonadotropin-Resistant Ovary Syndrome':ab,ti or 'Gonadotropin Resistant Ovary Syndrome':ab,ti or 'Resistant Ovary Syndrome':ab,ti or 'Premature Menopause':ab,ti or 'Decreased ovarian reserve':ab,ti or 'Diseased ovarian reserve':ab,ti or 'amenorrhea':ab,ti or 'Premature Menopause':ab,ti or 'Decreased ovarian reserve':ab,ti or 'Diseased ovarian reserve':ab,ti or 'ovarian function':ab,ti or 'Function Test, Ovarian':ab,ti or 'Function Tests, Ovarian':ab,ti or 'Ovarian Function Test':ab,ti or 'Test, Ovarian Function':ab,ti or 'Tests, Ovarian Function':ab,ti or 'Climacteric syndrome':ab,ti or 'Perimenopausal syndrome':ab,ti or 'Climacteric syndrome':ab,ti or 'Perimenopausal syndrome ':ab,ti

#2='Rat':ab,ti or 'Rattus':ab,ti or 'Rattus norvegicus':ab,ti or 'Rats, Norway':ab,ti or 'Rats, Laboratory':ab,ti or 'Laboratory Rat':ab,ti or 'Laboratory Rats':ab,ti or 'Rat, Laboratory':ab,ti or 'Animal Model':ab,ti or 'Animal Models':ab,ti or 'Model, Animal':ab,ti or 'Laboratory Animal Models':ab,ti or 'Animal Model, Laboratory':ab,ti or 'Animal Models, Laboratory':ab,ti or 'Laboratory Animal Model':ab,ti or 'Model, Laboratory Animal':ab,ti or 'Models, Laboratory Animal':ab,ti or 'Experimental Animal Models':ab,ti or 'Animal Model, Experimental':ab,ti or 'Animal Models, Experimental':ab,ti or 'Experimental Animal Model':ab,ti or 'Model, Experimental Animal':ab,ti or 'Models, Experimental Animal':ab,ti or 'Experimentation, Animal':ab,ti or 'Animal Research':ab,ti or 'Research, Animal':ab,ti or 'Animal Experimental Use':ab,ti or 'Animal Experimental Uses':ab,ti or 'Experimental Use, Animal':ab,ti or 'Experimental Uses, Animal':ab,ti or 'Animal Experiments':ab,ti or 'Animal Experiment':ab,ti or 'Experiment, Animal':ab,ti or 'Experiments, Animal':ab,ti or 'Animalia':ab,ti or 'Animal':ab,ti or 'Metazoa':ab,ti

**#3**='acupuncture':ab,ti OR 'electroacupuncture':ab,ti

**#4**= **#1 AND #2 AND #3**

**7.Cochrane library**

**#1**=(Polycystic Ovary Syndrome):ti,ab,kw OR (Ovary Syndrome, Polycystic):ti,ab,kw OR (Syndrome, Polycystic Ovary):ti,ab,kw OR (Stein-Leventhal Syndrome):ti,ab,kw OR (Stein Leventhal Syndrome):ti,ab,kw OR (Syndrome, Stein-Leventhal):ti,ab,kw OR (Sclerocystic Ovarian Degeneration):ti,ab,kw OR (Ovarian Degeneration, Sclerocystic):ti,ab,kw OR (Sclerocystic Ovary Syndrome):ti,ab,kw OR (Polycystic Ovarian Syndrome):ti,ab,kw OR (Ovarian Syndrome, Polycystic):ti,ab,kw OR (Polycystic Ovary Syndrome 1):ti,ab,kw OR (Sclerocystic Ovaries):ti,ab,kw OR (Ovary, Sclerocystic):ti,ab,kw OR (Sclerocystic Ovary):ti,ab,kw OR (Ovarian hyperstimulation syndrome):ti,ab,kw OR (Hyperstimulation Syndrome, Ovarian):ti,ab,kw OR (Hyperstimulation Syndromes, Ovarian):ti,ab,kw OR (Ovarian Hyperstimulation Syndromes):ti,ab,kw OR (Ovarian Hyperstimulation Syndrome, Familial Gestational Spontaneous):ti,ab,kw OR (Ovarian Insufficiency, Primary):ti,ab,kw OR (Ovarian Failure, Premature):ti,ab,kw OR (Premature Ovarian Failure):ti,ab,kw OR (Gonadotropin-Resistant Ovary Syndrome):ti,ab,kw OR (Gonadotropin Resistant Ovary Syndrome):ti,ab,kw OR (Resistant Ovary Syndrome):ti,ab,kw OR (Premature Menopause):ti,ab,kw OR (Decreased ovarian reserve):ti,ab,kw OR (Diseased ovarian reserve):ti,ab,kw OR (amenorrhea):ti,ab,kw OR (Premature Menopause):ti,ab,kw OR (Decreased ovarian reserve):ti,ab,kw OR (Diseased ovarian reserve):ti,ab,kw OR (ovarian function):ti,ab,kw OR (Function Test, Ovarian):ti,ab,kw OR (Function Tests, Ovarian):ti,ab,kw OR (Ovarian Function Test):ti,ab,kw OR (Test, Ovarian Function):ti,ab,kw OR (Tests, Ovarian Function):ti,ab,kw OR (Climacteric syndrome):ti,ab,kw OR (Perimenopausal syndrome):ti,ab,kw

**#2**=(Rat):ti,ab,kw OR (Rattus):ti,ab,kw OR (Rattus norvegicus):ti,ab,kw OR (Rats, Norway):ti,ab,kw OR (Rats, Laboratory):ti,ab,kw OR (Laboratory Rat):ti,ab,kw OR (Laboratory Rats):ti,ab,kw OR (Rat, Laboratory):ti,ab,kw OR (Animal Model):ti,ab,kw OR (Animal Models):ti,ab,kw OR (Model, Animal):ti,ab,kw OR (Laboratory Animal Models):ti,ab,kw OR (Animal Model, Laboratory):ti,ab,kw OR (Animal Models, Laboratory):ti,ab,kw OR (Laboratory Animal Model):ti,ab,kw OR (Model, Laboratory Animal):ti,ab,kw OR (Models, Laboratory Animal):ti,ab,kw OR (Experimental Animal Models):ti,ab,kw OR (Animal Model, Experimental):ti,ab,kw OR (Animal Models, Experimental):ti,ab,kw OR (Experimental Animal Model):ti,ab,kw OR (Model, Experimental Animal):ti,ab,kw OR (Models, Experimental Animal):ti,ab,kw OR (Experimentation, Animal):ti,ab,kw OR (Animal Research):ti,ab,kw OR (Research, Animal):ti,ab,kw OR (Animal Experimental Use):ti,ab,kw OR (Animal Experimental Uses):ti,ab,kw OR (Experimental Use, Animal):ti,ab,kw OR (Experimental Uses, Animal):ti,ab,kw OR (Animal Experiments):ti,ab,kw OR (Animal Experiment):ti,ab,kw OR (Experiment, Animal):ti,ab,kw OR (Experiments, Animal):ti,ab,kw OR (Animalia):ti,ab,kw OR (Animal):ti,ab,kw OR (Metazoa):ti,ab,kw

**#3**=(acupuncture):ti,ab,kw OR (electroacupuncture):ti,ab,kw

**#4**= **#1 AND #2 AND #3**

**8.Web of science**

**#1**= (Polycystic Ovary Syndrome OR Ovary Syndrome, Polycystic OR Syndrome, Polycystic Ovary OR Stein-Leventhal Syndrome OR Stein Leventhal Syndrome OR Syndrome, Stein-Leventhal OR Sclerocystic Ovarian Degeneration OR Ovarian Degeneration, Sclerocystic OR Sclerocystic Ovary Syndrome OR Polycystic Ovarian Syndrome OR Ovarian Syndrome, Polycystic OR Polycystic Ovary Syndrome 1 OR Sclerocystic Ovaries OR Ovary, Sclerocystic OR Sclerocystic Ovary OR Ovarian hyperstimulation syndrome OR Hyperstimulation Syndrome, Ovarian OR Hyperstimulation Syndromes, Ovarian OR Ovarian Hyperstimulation Syndromes OR Ovarian Hyperstimulation Syndrome, Familial Gestational Spontaneous OR Ovarian Insufficiency, Primary OR Ovarian Failure, Premature OR Premature Ovarian Failure OR Gonadotropin-Resistant Ovary Syndrome OR Gonadotropin Resistant Ovary Syndrome OR Resistant Ovary Syndrome OR Premature Menopause OR Decreased ovarian reserve OR Diseased ovarian reserve OR amenorrhea OR Premature Menopause OR Decreased ovarian reserve OR Diseased ovarian reserve OR ovarian function OR Function Test, Ovarian OR Function Tests, Ovarian OR Ovarian Function Test OR Test, Ovarian Function OR Tests, Ovarian Function OR Climacteric syndrome OR Perimenopausal syndrome

**#2**= Rat OR Rattus OR Rattus norvegicus OR Rats, Norway OR Rats, Laboratory OR Laboratory Rat OR Laboratory Rats OR Rat, Laboratory OR Animal Model OR Animal Models OR Model, Animal OR Laboratory Animal Models OR Animal Model, Laboratory OR Animal Models, Laboratory OR Laboratory Animal Model OR Model, Laboratory Animal OR Models, Laboratory Animal OR Experimental Animal Models OR Animal Model, Experimental OR Animal Models, Experimental OR Experimental Animal Model OR Model, Experimental Animal OR Models, Experimental Animal OR Animal Research OR Experimentation, Animal OR Research, Animal OR Animal Experimental Use OR Animal Experimental Uses OR Experimental Use, Animal OR Experimental Uses, Animal OR Animal Experiment OR Animal Experiments OR Experiment, Animal OR Experiments, Animal OR Animalia OR Animal OR Metazoa

**#3**= acupuncture OR electroacupuncture)

**#4**= **#1 AND #2 AND #3**
